# Recent advances in quantitative LA-ICP-MS analysis: challenges and solutions in the life sciences and environmental chemistry

**DOI:** 10.1007/s00216-015-8858-0

**Published:** 2015-07-14

**Authors:** Andreas Limbeck, Patrick Galler, Maximilian Bonta, Gerald Bauer, Winfried Nischkauer, Frank Vanhaecke

**Affiliations:** Institute of Chemical Technologies and Analytics, Division of Instrumental Analytical Chemistry, TU Wien, Getreidemarkt 9/164, 1060 Vienna, Austria; Elkem AS, Technology, Central Analytical Laboratory, Fiskaaveien 100, P.O. Box 8040, Vaagsbygd, 4675 Kristiansand Norway; Department of Analytical Chemistry, Ghent University, Krijgslaan 281 - S12, 9000 Ghent, Belgium

**Keywords:** LA-ICP-MS, Quantitative analysis, Certified reference material, Matrix-matched standards, Internal standard correction, Liquid standards

## Abstract

Laser ablation–inductively coupled plasma–mass spectrometry (LA-ICP-MS) is a widely accepted method for direct sampling of solid materials for trace elemental analysis. The number of reported applications is high and the application range is broad; besides geochemistry, LA-ICP-MS is mostly used in environmental chemistry and the life sciences. This review focuses on the application of LA-ICP-MS for quantification of trace elements in environmental, biological, and medical samples. The fundamental problems of LA-ICP-MS, such as sample-dependent ablation behavior and elemental fractionation, can be even more pronounced in environmental and life science applications as a result of the large variety of sample types and conditions. Besides variations in composition, the range of available sample states is highly diverse, including powders (e.g., soil samples, fly ash), hard tissues (e.g., bones, teeth), soft tissues (e.g., plants, tissue thin-cuts), or liquid samples (e.g., whole blood). Within this article, quantification approaches that have been proposed in the past are critically discussed and compared regarding the results obtained in the applications described. Although a large variety of sample types is discussed within this article, the quantification approaches used are similar for many analytical questions and have only been adapted to the specific questions. Nevertheless, none of them has proven to be a universally applicable method.

## Introduction

Laser ablation (LA) in combination with inductively coupled plasma–mass spectrometry (ICP-MS) is a powerful technique for the direct elemental analysis of solid samples. This technique provides major, minor, and trace element information with a wide elemental coverage, excellent limits of detection, and a linear dynamic range of up to 10 orders of magnitude, while also enabling microanalysis, depth profiling analysis, and 2-dimensional elemental mapping. Further advantages of LA-ICP-MS are minimal sample preparation, high sample throughput, access to isotopic information, and the possibility of analyzing both conductive and non-conductive and opaque and transparent materials [1–4].

However, two fundamental aspects of processes involved constrain the ability of LA-ICP-MS to act as a universal method for direct analysis of solid samples. The first major drawback of LA-ICP-MS is that the abundances of the ions detected after *m*/*z* separation are often not entirely representative of the composition of the original sample. In the literature, this problem is often referred to as “elemental fractionation” [5, 6], although this term is also used to describe time-dependent changes in the composition of the ion beam in the mass spectrometer. Besides the ablation process itself (e.g., non-stoichiometric effects due to the preferred ablation of more volatile compounds), the transport of the aerosol particles from the ablation chamber into the ICP (e.g., differences in gravitational settling between smaller and larger particles) and vaporization, atomization, and ionization in the ICP (less efficient for larger particles) are also important contributors to fractionation effects. A detailed discussion of the individual contributions to elemental fractionation and the strategies developed for minimizing the influence exerted can be found in the literature [7–14].

The second major problem connected with the use of LA-ICP-MS for direct analysis of solid samples is the difference in the interaction between the laser beam and the sample surface observed for various matrices, causing changes in the mass of analyte ablated per pulse due to differences in the properties of the matrices investigated (e.g., absorptivity, reflectivity, and thermal conductivity). The aerosol particles produced during ablation of different matrices may vary in size and geometry, thus having an effect on the sample transport efficiency from the ablation cell to the plasma [15]. Both effects contribute to differences in the mass load of the plasma and give rise to matrix effects, since the vaporization, atomization, and ionization efficiencies of the analytes introduced into the plasma depend on the mass load [16]. Sample-related “matrix effects” therefore jeopardize the accuracy of LA-ICP-MS analysis and complicate quantification [2–4, 17–20].

**Fig. 1:**
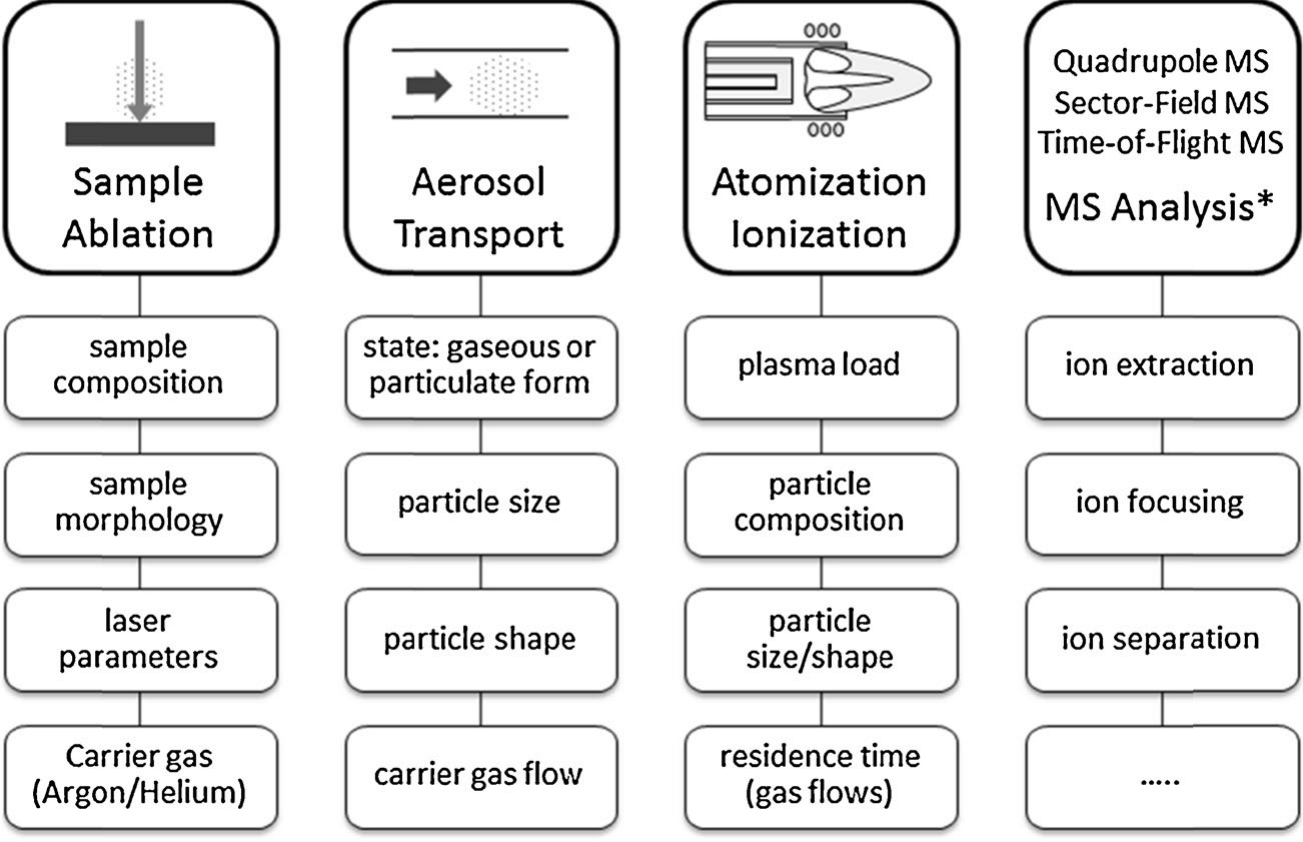
Sources of error in LA-ICP-MS analysis, * not discussed within this review

As a result, elemental fractionation and matrix effects occur simultaneously, leading to LA-ICP-MS signals that are not representative of the elemental composition of the sample investigated. The sensitivity or absolute signal intensity can vary significantly for samples with the same analyte concentrations, but different matrix compositions and/or physical properties. At this point, it has to be mentioned that mass spectrometric separation and detection of the ions generated can also contribute to the bias in LA-ICP-MS results. However, an explanation of the corresponding sources of bias is beyond the scope of this work; details on these issues can be found in a recently published review article [21]. Figure 1 schematically summarizes the individual steps of LA-ICP-MS analysis prone to elemental fractionation and matrix effects.

As a consequence of the increasing interest in the use of LA-ICP-MS in various scientific fields, research has been devoted to overcoming the aforementioned drawbacks. In the few last years, attempts were made to address the limitations of LA-ICP-MS by improving the instrumental parameters relevant to aerosol formation. Most of this work focused on the influence of the wavelength of the laser radiation (especially important for transparent materials) and the pulse duration (especially important for metallic samples). With the use of shorter ultraviolet wavelengths and pulse durations in the femtosecond (fs) range, instead of the nanosecond range, a significant reduction of elemental fractionation and matrix effects is enabled. Furthermore, the laser beam profiles were changed from Gaussian to (pseudo) flat-top profiles, leading to optimized ablation performance. However, complete elimination of these effects is still not possible. Ongoing research is therefore dedicated to methodological developments that permit correct quantification with the currently available instrumentation for LA-ICP-MS analysis.

The purpose of this review is to summarize state of the art procedures and recent developments in quantitative LA-ICP-MS analysis of samples originating from the fields of life sciences and environmental chemistry. In addition to traditional approaches, novel concepts for the preparation of matrix-matched standards, such as the deposition of elemental coatings or thin polymeric films containing an internal standard on the sample surface, as well as quasi-simultaneous measurement of standard and sample using a spinning platform will be presented. Capabilities and limitations of the different approaches will be compared, critically examined, and evaluated on the basis of their suitability for general use.

## Common concepts for quantification in LA-ICP-MS

Even though the application range of LA-ICP-MS is wide and the sample types analyzed are various, some approaches for quantification are applied to a large variety of sample types. The basic principles of the methods described below are the same, while modifications thereof will be presented in the sections dedicated to specific sample types.

External calibration utilizing certified reference materials (CRMs) which match the composition of the sample to be investigated to the largest possible extent—preferably exactly—is the most reliable method for accurate quantification in LA-ICP-MS [20, 22–24]. If this prerequisite is met, ablation, transport, atomization, and ionization of sample and standard can be considered to be (nearly) identical, enabling reliable quantification. For each CRM, a detailed certificate is available containing information regarding component concentrations. Additionally, in the literature, preferred concentration values are available for non-certified sample constituents [25]. However, the lack of appropriate CRMs for the majority of sample types (in particular for samples from environmental, biological, or medical origin) limits the applicability of this approach. Thus, alternative quantification strategies are mandatory.

A promising approach for quantification is the preparation of matrix-matched calibration standards, prepared from material with the same matrix as the sample [26–29]. Procedures for sample preparation preceding LA-ICP-MS analysis reported in the literature include fusion with borate, embedding in a polymer resin, or preparation of a pressed disk in the presence of a binder. Benefits of these sample preparation approaches are that they facilitate the addition of one or more internal standards, known amounts of the analyte(s) of interest (for standard addition purposes) or isotopically enriched spikes (for isotope dilution purposes), as well as the possibility to adjust the analyte concentrations as required (dilution). A major drawback is the applicability to powdered samples only; samples that are compact in their native form require additional sample pretreatment (i.e., milling, grinding). Furthermore, it has to be considered that this type of adjustment of the sample matrix is automatically accompanied by analyte dilution, which decreases the detection power of the analysis approach.

A frequently applied method in combination with external calibration (i.e., CRMs and in-house standards) is signal normalization to an internal reference or internal standard [30–33]. This approach can be used to further improve the accuracy of the quantification results, since the influence of remaining differences between sample and standard can be minimized. Variations in sample ablation and transport as well as ICP-related alterations in signal intensity (e.g., changing plasma conditions) can be corrected for using an internal standard. A precondition for the successful application of this method is that the internal standard element and the analyte element are homogeneously distributed within the sample, and that their behavior during ablation, transport, and ionization is similar. In this course, crucial parameters are, e.g., the form in which the element is transported from the ablation cell into the ICP (gaseous or particulate) and its mass and ionization potential. The element being used as internal standard can either occur naturally in the sample or is added during the sample preparation process. Optimally, the concentration of the element used as internal standard in the sample is known. However, for successful application it is sufficient that the concentrations in the standard and sample are equal.

“Solid–liquid” calibration in which a dual flow system allows simultaneous introduction of a nebulized aqueous standard solution and laser ablated material is an attractive alternative to the use of matrix-matched solid standards [3, 34, 35]. In this procedure, the carrier gas flow coming from the ablation cell is mixed with an aerosol generated by nebulization of an aqueous standard solution. Besides the addition of aerosol to the gas lines leading to the ICP [36], the use of micronebulizers has also been proposed to add the aerosol to the gas flow directly at the site of material ablation [36, 37]. Not only standards with natural isotopic composition [36] but also isotopically enriched standards [37] have been used for such experiments. Standard and blank solution are alternately added to the sample stream, such that the accompanying difference in signal intensity can be used to quantify the analyte concentrations in the sample. A correction for the differences in ablation efficiency is required and when aiming to maintain the advantages of “dry plasma” conditions, such as a reduced level of oxide interferences, the wet aerosol must be desolvated prior to its mixing with the sample aerosol. However, for special applications, wet plasma conditions may also offer improved measurement conditions, e.g., in terms of signal stability [38]. This method enables quantification based on aqueous standards, and can compensate for matrix-related ionization differences. However, possible variations in ablation efficiency or altered transport efficiencies cannot be accounted for.

Although the quantification approaches mentioned above have been successfully applied in several research fields, including material sciences, geo- and cosmochemistry, environmental chemistry, biology, and medicine [3, 4, 14, 19, 22, 23, 39], their successful application to any kind of sample is not guaranteed. Thus, further improvements are required, which could be achieved either by reducing the extent of matrix effects by using optimized instrumental parameters (e.g., laser radiation wavelength, pulse duration, robustness of ICP) or by developing alternative strategies for quantification.

## Analysis of hard tissues and compact samples

Naturally occurring compact materials, such as rocks and minerals, bones, teeth, claws, feathers, or nails, require no pretreatment like milling or pressing prior to LA-ICP-MS analysis. However, often it might be necessary to flatten the exposed sample surface using a grinding and/or polishing step. Within this review, only samples of biological origin will be discussed. Samples of geological origin will not be described in detail here; for this kind of samples, detailed information can be found in journals devoted to geology and geochemistry.

While quantitative determination of major, minor, and trace elements in the samples mentioned above is the main field of application of LA-ICP-MS, the technique also offers the possibility of performing spatially resolved analysis, which is of interest for studying element distributions (mapping or imaging and depth profiling analysis) or inhomogeneities (solid or fluid inclusions) in many materials. Applications solely dedicated to visualizing elemental distributions are also beyond the scope of this paper, which focuses on those applications in which estimation of bulk or local concentrations forms at least part of the investigation and, possibly, some effort is made for cross-validation using an alternative analytical approach. Yet, calibration approaches discussed here can self-evidently also be deployed in mapping or imaging applications.

### Biogenic carbonates: calibration

Although LA-ICP-MS lends itself specifically well to spatially resolved analysis, its application for bulk analysis is justified in cases where a dedicated area across a given set of samples has to be reproducibly analyzed in situ in order to enable comparison. This has been specifically exploited for the investigation of fish otoliths at their cores and edges for the purpose of origin determination of fish populations [31, 40–47], or for the investigation of changes in the prevailing conditions, reflected in the microchemistry of very narrow otolith bands [32, 48–50]. The daily accretion of calcium carbonate layers in otoliths and their permanent retention of chemical fingerprints in the form of various elemental impurities make them an ideal target for this type of investigation [51]. Similar incremental growth behavior and chemical matrix are found in mussel shells and corals. Combined with their immobility, it makes these objects valuable environmental monitors [26, 27, 52–54]. Also larvae tracking applications have been reported for mussels [55, 56]. This type of investigation can be summarized under the term *sclerochronology*.

The different NIST SRM glasses 610, 612, and 614 (National Institute of Standards and Technology, Gaithersburg, USA), with certified trace element concentrations over approximately three orders of magnitude, from the low microgram per gram level to hundreds of micrograms per gram, are by far the most frequently used materials for calibrating biogenic calcium carbonate measurements by LA-ICP-MS [31, 32, 40–43, 46, 48–50, 52–56]. Trying to improve the analytical results, Arkhipkin et al. compensated for the difference in matrix composition between the NIST SRM glass and biogenic calcium carbonate via the introduction of in-house correction factors [46]. However, according to Jochum et al., calibration using NIST SRM glasses as such already results in accurate values for the refractory elements, whereas a closer matrix-matching using calcium carbonate pellets has to be applied for low boiling point elements, such as Pb [53]. Another decisive parameter for measurement accuracy when using the NIST SRM glasses for calibration is the set of reference concentrations used for these materials [53, 57, 58]. Custom-made fused glasses are an alternative to the NIST SRM glasses for calibration purposes. Such glasses were prepared by Sinclair et al. by blending biogenic carbonate (coral powder) with silica in a ratio of 1:1, followed by fusion at 1650 °C and by Perkins et al. by blending Li_2_B_4_O_7_ in excess with synthetic CaCO_3_, MgO, and gravimetric additions of the analytes of interest, followed by fusion over a burner flame [26, 27]. Sinclair et al. obtained reference concentrations for their glasses using solution-based isotope dilution either by ICP-MS or thermal ionization mass spectrometry for all elements other than B, which was calibrated via LA-ICP-MS against NIST SRM 612 using B concentrations from the literature [26].

Besides NIST SRM glasses, carbonate pellets represent the second largest group of calibration materials in the field of biogenic carbonate analysis by LA-ICP-MS [27, 44, 45, 47, 52, 53, 59]. Different carbonate materials pressed into pellets have been used, including the commercially available synthetic calcium carbonates USGS MACS-1 and MACS-3 (United States Geological Survey, Reston, VA, USA) [44, 52, 53], fish otolith powder NRC FEBS-1 (National Research Council Canada, Ottawa, Canada) [47], synthetic in-house carbonates prepared by co-precipitation [45], or in-house standards prepared from gravimetric blends of the analytes of interest and either commercially available calcium carbonate or crushed biogenic carbonate [27]. In one case, the authors blended NIST SRM glasses and carbonate pellets to obtain calibration standards, without revealing whether all materials were used in the same calibration function [52]. However, despite the multitude of calibration materials obviously at hand, the fact that otoliths also contain a significant amount of organic matter is commonly not accounted for [27, 51].

Without exception, all authors use Ca as internal standard element for normalization, albeit with little agreement regarding their choice of the Ca nuclide used for this purpose. This is mainly a question of user experience, instrumental sensitivity, and the means available to overcome spectral interference. All Ca isotopes, apart from the most abundant (^40^Ca) and the least abundant (^46^Ca), have been reported in papers related to LA-ICP-MS analysis of biogenic calcium carbonates and referenced in this review. There is also a fair amount of disagreement with respect to the Ca concentration assumed or measured for otoliths, which is critical for obtaining accurate data. Whereas some authors calculate a theoretical Ca concentration based on CaCO_3_ stoichiometry [32, 40, 41, 45, 48, 50], others measure it in advance using conventional solution nebulization ICP-MS or ICP-OES [31, 47]. As a result, reported Ca concentrations range from 35 to 40 %. Even when estimates are based on CaCO_3_ stoichiometry only, some disagreement is possible.

### Biogenic hard tissues: claws, feathers, fish scales, and hair: calibration

LA-ICP-MS also becomes an asset when minimally invasive sampling and analysis are required. LA-ICP-MS has been used for the quantitative analysis of animal claws, feathers, fish scales, snake tail clippings, animal hair, human hair, and human finger nails [60–71].

Ethier et al. and Kaimal et al. used the NIST SRM 612 glass as a standard in the context of multi-element analysis of badger claws and bird feathers, respectively [60–62]. Both teams used concentration data obtained via LA-ICP-MS for statistical classification of their results. Whereas Ethier et al. used S as an internal standard as a consequence of the high cysteine content of the sample matrix keratin [60, 61], Kaimal et al., used ^42^Ca, assuming a homogeneous Ca distribution [62]. For Ethier et al., the use of S as internal standard, quantified in advance using conventional solution nebulization ICP-MS, required the introduction of inter-element sensitivity factors, established from the ICP-MS mass response curve obtained upon ablation of NIST SRM glass. The authors indicated that this approach only yields semiquantitative data. Human and animal hair, as well as human finger nails or clippings thereof, all predominantly comprised of keratin, have been the subject of many studies owing to their capability as a biomonitor of past (trace) element exposure [66–70]. The application of in-house hair or nail material for calibration is quite commonplace in this context [66–70]. Rodushkin and Axelsson used in-house finger nail material, powdered and pressed into a pellet, for calibration of finger nail measurements [70]. Reference concentrations were obtained from conventional solution nebulization ICP-MS after sample digestion. For hair analysis, the certified GBW07601 hair reference material (Institute of Geophysical and Geochemical Exploration, Lanfang, China) was used for calibration [70]. Similarly, Stadlbauer et al. used BCR CRM 397 hair reference material (Institute for Reference Materials and Measurements, Geel, Belgium), pressed into a pellet with polyethylene as a binder [71]. Bartkus et al. and Arriaza et al. both used whole in-house hair standards, quantified for Pb and As by conventional solution nebulization and hydride generation ICP-MS, for calibration of LA-ICP-MS measurements [68, 69]. Dressler et al. pursued calibration of LA-ICP-MS measurements of mouse and human hair by simultaneous aspiration of multi-element solutions (at several concentration levels) via a conventional nebulizer [66]. The wet aerosol was mixed on-line with the dry aerosol coming from the ablation chamber in the injector tube of the ICP torch. Differences in aerosol generation and transport efficiencies between solution nebulization and LA were assessed by ablating in-house hair material with known analyte element concentrations. The in-house hair standard material was prepared by immersion of hair strands in a multi-solution, subsequent drying, and digestion of the material thus obtained for the determination of reference concentrations via conventional solution nebulization ICP-MS. Sela et al. used a similar approach, but one based on standard addition using an ultrasonic nebulizer equipped with a desolvation unit [67]. The dry aerosol intended for calibration was directed through the ablation cell for mixing with the LA aerosol before introduction into the ICP. Concentrations were determined for single hair strands and hair powder, both fixed on carbon tabs. As for the internal standard, both ^32^S and ^34^S have been used for hair and finger nail samples [66, 67, 70]. Rodushkin and Axelsson report S concentrations of 4.77 ± 0.41 % and 3.30 ± 0.56 % for hair and finger nails, respectively [70]. S concentrations were obtained on the basis of hair and finger nail samples of approximately 100 Swedish individuals. Stadlbauer et al. used a quadrupole-based ICP-MS instrument equipped with a reaction cell and adopted sulfur in the form of (^32^S^16^O)^+^ as internal standard to avoid spectral overlap of the ^32^S^+^ peak with that from the oxygen dimer ion ^16^O_2_^+^ at *m*/*z* = 32 [71]. Also ^13^C has been reported as an internal standard for LA-ICP-MS analysis [68, 69]. However, the use of ^13^C as internal standard is associated with some major drawbacks. Those will be described in the chapter ‘measurement of soft tissues and protein samples’ in the section ‘internal standards’ in more detail.

Holá et al. and Flem et al. both developed LA-ICP-MS methods for trace element quantification in fish scales as an alternative to otolith sampling [63, 64]. Since fish scales contain (Ca-deficient) hydroxyapatite, Holá et al. used NIST SRM 1486 bone meal for external calibration [63]. In contrast, Flem et al. used not less than six different glass reference materials for calibration, namely NIST SRMs 610, 612, 614, 616, NIST SRM 1830 soda limestone float glass, and USGS TB-1 basaltic glass [64]. TB-1 was only introduced for calibration of Sr. Flem et al. commented that for the purpose of their study, normalized data only would have sufficed, yet calibration against the different glasses was included in order to be able to at least provide concentration estimates for later use. Both groups used Ca as an internal standard, determined by electron microprobe analysis in both cases [63, 64]. Flem et al. quote an average Ca concentration of 37.4 ± 0.4 % for a set of fish scales [64], whereas Holá indicate Ca concentrations for three line scans on one fish scale ranging from 23.5 to 26.5 % [63]. Holá et al. also gave some indication of the homogeneity of Ca in fish scales through a spatial distribution map obtained by electron microprobe analysis [63]. Alternatively, calibration using spiked hydroxyapatite prepared as in-house calibration materials has been reported for LA-ICP-MS investigations of (human) bone and teeth [71].

The last example given describes the direct analysis of water snake tail clippings by LA-ICP-MS as an ecotoxicology tool [65]. Given the complexity of the sample material containing inorganic bone, calcium carbonate, muscle blood, and skin, Jackson et al. resorted to in-house preparation of matrix calibration standards from water snake tail sample material [42]. Reference concentrations were obtained from conventional solution nebulization ICP-MS and ^13^C was used as internal standard for LA-ICP-MS.

### Validation

It is not uncommon to omit validation from the analytical procedure entirely, which may be justified in cases where consistency of results is more important than accuracy, such as in statistical classification of the samples analyzed among different groups [40, 42, 43, 46, 62, 64]. In cases where analyte concentrations are obtained via LA-ICP-MS using non-matrix-matched standards, one should refrain from comparing results to other sets of data obtained for the same sample type by a different analytical approach without validating the quantitative results. Several approaches for this purpose were reported in the literature. Validation by re-measuring the calibration standard, in this case NIST SRM 610 glass, as a sample has also been described, but this is clearly a far from ideal assessment of measurement accuracy [32]. This is appreciated by some authors through the introduction of a reference material as an unknown in the analytical protocol. Different reference materials including USGS MACS-1 and MACS-3 synthetic calcium carbonate, NIES-022 fish otolith powder (National Institute for Environmental Studies, Tsukuba, Japan), and NRC FEBS-1 fish otolith powder have been used for this purpose, as have the limestone reference materials GSJ JLS-1 (Geological Survey of Japan, Tsukuba, Japan), GSR-6 (Ministry of Land and Resources, Beijing, China), and BAS CRM-393 (Bureau of Analysed Samples Ltd, Middlesbrough, UK) [27, 41, 44, 47, 53].

Some authors also validated their LA-ICP-MS results by conventional solution nebulization ICP-MS. Phung et al. performed LA-ICP-MS analysis in holes left by micro-drill sampling for solution nebulization ICP-MS analysis and subsequently compared results from both procedures involving two different LA-ICP-MS facilities [52]. Results agree generally within the quoted analytical errors, with a few exceptions depending on the hole analyzed. Sinclair et al. converted LA-ICP-MS line scans on corals into average concentrations for five elements and compared these to results from solution nebulization ICP-MS of a digest of the same sample [26]. Deviations ranged from approximately −3 to 30 % between the two methods. Dressler et al., Sela et al., and Rodushkin and Axelsson all compare their LA-ICP-MS results for human hair and nail samples to results from conventional solution nebulization ICP-MS [66, 67, 70]. Dressler et al. achieve agreement within analytical error [66], and the results of Rodushkin and Axelsson also showed a good correlation of LA-ICP-MS and solution nebulization ICP-MS results; generally, LA-ICP-MS results are within 30 % of solution nebulization ICP-MS results. Average LA-ICP-MS results obtained by Holá et al. for fish scales are generally higher than the corresponding solution nebulization ICP-MS data, which is also explained by the complexity of the sample matrix, i.e., analyte enrichment in the uppermost layer ablated from fish scales [63].

There is a general agreement between the authors regarding the use of gas blanks for baseline correction. Typically, limits of detection are calculated from three or ten times the standard deviation of the gas blank, divided by the slope of the calibration line or the instrumental sensitivity [31, 42, 44–46, 48]. However, as discussed by Rodushkin and Axelsson, detection limits in LA-ICP-MS depend on the volume of ablated material, the analyte mass, the ionization energy of the analyte, its isotopic abundance, and the ion transmission efficiency [70]. Aerosol size distribution and transport efficiency to the ICP-MS presumably play a role too. For the analysis of hair and nail, detection limits in LA-ICP-MS range from picograms per gram to nanograms per gram and are quoted as only marginally nferior to conventional solution nebulization ICP-MS as a result of sample dilution after digestion for the latter approach.

## Measurement of soft tissues and protein samples

In recent years, LA-ICP-MS has also become a technique of growing interest in the life sciences. The effect of variations in trace elemental concentrations, and especially metal–protein interactions are increasingly studied in biological and biomedical investigations [72]. The sample types reported on vary from native samples, such as plant material [73–76], and thin sections of animal/human body tissues (e.g., liver [77], brain [78–80], eye tissue [81], kidney [82], and others), to electrophoretically separated metalloproteins [83, 84]. Typically, natural element distributions within the biological samples were investigated. In special cases, the use of isotopic analysis has been reported (e.g., as tracers for metal uptake in organisms). For example, Florez et al. exposed *Daphnia magna* to isotopically enriched Zn tracers and produced isotope ratio images with 30-μm spatial resolution [85]. A more detailed description of possibilities and limitations of isotopic analysis can be found elsewhere [86].

Like in every other field of LA-ICP-MS applications, quantification is a crucial aspect. Problems aggravating reliable quantification are the large variety of sample types and properties, as well as a lack of suitable standard materials. However, especially when thin sections of sample material are used (preparation of thin sections is common practice in the medical sciences), some aspects of quantification are facilitated, and some new issues may arise. Those thin-cuts typically have thicknesses of 5–20 μm, thus providing the opportunity to ablate the sample material (the entire depth) completely with a few laser shots, i.e., during one cycle of analysis. The analysis of thin layers renders the analysis of tissue sample much easier, since differences in penetration depth of the laser beam into the sample material do not need to be considered. This gives the opportunity of applying thin layers containing a standard for signal normalization above or below the sample. Those also have to be completely ablated along with the sample. Independently from the quantification strategy used, reliable quantitative analysis is only possible when the thin sections of the samples have equal thicknesses. Some quantification approaches that will be described in later sections rely on this assumption as they cannot compensate for varying layer thickness. If the tissue thicknesses are varying within one sample or the whole tissue sample can not be ablated in a single run, i.e., for thicker samples or bulk material, internal standardization has to be used as for many other types of samples described in this article.

### Quantitative imaging approaches

A large number of LA-ICP-MS applications involving soft tissue samples aim to unravel the 2-dimensional trace element distributions (bioimaging or mapping) [87–89]. To preserve information on the spatial analyte distribution, tissue samples are analyzed as thin slices without any prior homogenization step. Three major problems have to be addressed to ensure reliable analysis results: material ablation and aerosol transport are highly matrix-dependent, the efficiency and location of the ionization process in the ICP are a function of the particle size distribution of the aerosol produced via LA [24], which, similarly, is matrix-dependent, and during the measurement time, instrument instability and signal drift may occur because of changing experimental conditions (e.g., cone conditions, vacuum pressure). As a result of those factors, even the measurement of reliable qualitative distribution maps is not self-evident; ensuring reliable quantitative results is even more challenging. Not all approaches that have been presented in the past are capable of adequately addressing the complete range of limitations mentioned.

#### Solid standard materials

Another method for quantification is the use of solid standard materials. Thereby, a suitable standard material can be manufactured for almost every sample type. CRMs for tissues are only scarcely available, and are in most cases not compatible with the specific experimental conditions (e.g., tissue types, trace elements selected, and/or concentration range).

While quantitative elemental analysis of homogenous materials using LA-ICP-MS can often rely on matrix-matched standards, biological samples may significantly differ in their composition even within several sections of a single sample, and therefore different methods are needed to ensure accurate quantification. Various approaches have been proposed and used to facilitate and improve the quantification of trace elemental distributions in biological tissues and to overcome the problem of often pronounced sample inhomogeneity. Even though many alternative methods for quantification have been proposed, the classical and still most often used method is the use of matrix-matched standards [90]. Those have to be prepared in-house and tuned to the specific application. The preparation of matrix-matched tissue standards has been described by Hare et al. [90] in detail. In short, the selected tissue is homogenized and spiked with an aqueous standard containing the elements of interest; the spiking process is performed at different concentrations enabling the determination of calibration functions. An aliquot of the homogenized and spiked tissues is acid digested for determining the actual analyte element concentrations in the standards. After freezing of the standard, a cryo-cut of desired thickness is prepared for LA-ICP-MS measurement. Alternative approaches for quantification in LA-ICP-MS analysis of biological tissue using solid standards aim at rendering the process of standard preparation easier. Approaches to facilitate the manufacturing process of standard materials use gelatin [91], agarose gel [92], or sol-gel [93] standards, spiked with appropriate amounts of the elements of interest. The preparation of those materials is similar to that of matrix-matched tissue standards, but less tedious. The goal is to minimize the handling of biological materials and still end up with standards with a similar matrix composition (mostly in terms of carbon content and density). One approach uses a polymeric film, spiked with the elements of interest, applied to a glass slide before attaching a thin section of the sample [94]. Assuming simultaneous ablation of standard and sample material, this approach will lead to correction for matrix effects during the measurement—similar to a single standard addition approach. Another way to facilitate standard preparation for analysis of biological tissues is printing of standards onto paper using a commercially available office inkjet printer [95, 96]. Conventional paper can be used for standard preparation, and the inks may be spiked with elements of interest. This method has been successfully used to quantify trace elements in different biomaterials. Reifschneider et al. proposed a method to reduce matrix effects by embedding biological tissues into epoxy resins [97]. In the embedding method used, complete penetration of the resin into the tissue material was ensured. The standards are prepared from epoxy resins without embedded tissue; as the main matrix material is the epoxy resin, no major difference in matrix composition exists between standards and samples. All methods discussed so far try to simulate the ‘average’ matrix conditions in the tissues presented. But, a biological sample can be very inhomogeneous and matrix compositions may vary significantly, even within a single sample. Local variations in the matrix composition can lead to inaccurate quantitative results because of alterations in material ablation, aerosol transport, and analyte ionization efficiencies. The approaches for quantification described can reduce such matrix-related effects on the ablation and analyte ionization. Still, those approaches offer no possibility for the correction of instrument instability and/or signal drift or for the reduction of signal variations originating from inhomogeneities in the sample matrix.

#### Internal standards

Similar to conventional solution ICP-MS measurements, an internal standard can help to correct for changes in the signal intensity originating from instrument instability and/or signal drift. During the long measurement times of imaging experiments (usually 4–30 h), gas flow rates, cone and vacuum conditions, and other experimental conditions may vary. Furthermore, the pronounced inhomogeneity of the samples investigated with profoundly changing matrix compositions require the use of (an) internal standard(s) for reliable quantification [98]. In most publications, carbon has been proposed as the internal standard, as it is abundant in every biological sample and is often uniformly distributed across the sample. However, it has been shown that carbon is not an optimal internal standard [99], as its ionization potential is significantly higher than those of commonly investigated elements, such as most transition metals, and an altered carbon load in the plasma may change the ionization efficiency of some of the analytes monitored substantially. Furthermore, the transport of carbon into the ICP can partly occur in the form of carbon dioxide, which will lead to transport properties and efficiencies that can markedly differ from those elements that are transported as particulate matter only [99]. Therefore, normalization to carbon as internal standard may lead to distortions of the actual analyte distribution, causing inaccurate quantification results. Sulfur has also been proposed as a sample-inherent internal standard [77]; however, it is not evenly distributed in most tissues and, because of its high first ionization energy, similar problems as mentioned for carbon may be expected. Therefore, alternative approaches for signal normalization have been developed to improve the existing quantification methods. Both online addition of wet aerosol [36, 37] and the method with the spiked polymer layers [94] or the epoxy resin [97] have used signal normalization as part of the quantification approach. However, in the first two approaches mentioned, no internal standard in the traditional meaning was used, as ablation of sample and internal standard take place subsequently, and not simultaneously. Another way to normalize the analyte signal which was proposed by Konz et al. [100] and shown to be feasible for quantification [81, 95] is the sputtering of the samples with a thin gold layer; however, this approach only provides a pseudo-internal standard for the same reason. Only the epoxy embedding method provides a true internal standard. The tissue samples are immersed in the epoxy resin containing the internal standard; the resin completely penetrates the tissue material. Therefore, the internal standard is ablated simultaneously with the sample material. Combining the preparation of external standards with matrices similar to the sample material with the use of an internal or pseudo-internal standard can counteract the matrix-related effects on the material ablation, aerosol transport, and analyte ionization, as well as that of instrument instability and/or signal drift.

**Fig. 2:**
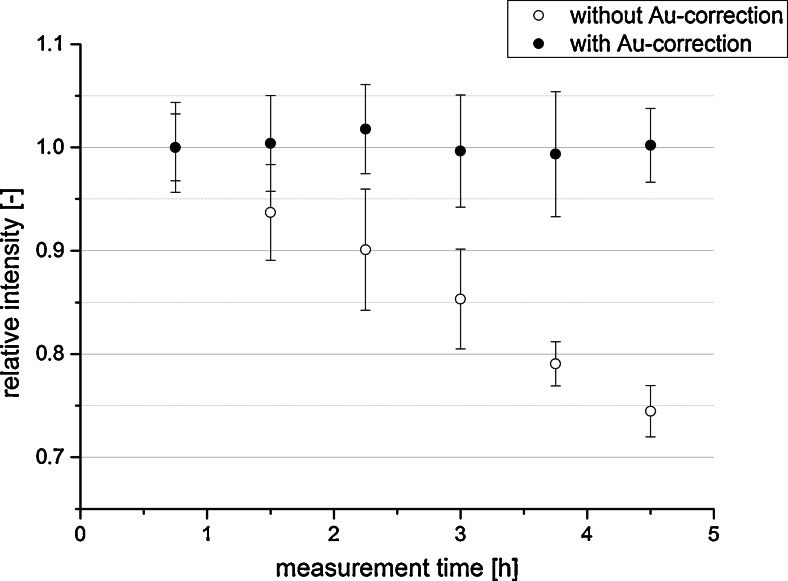
Signal intensities at different time points with and without gold normalization; averages of 25 data points are displayed (*n* = 25)

The necessity of instrumental drift correction was described by Hare et al. [20] and shown by Bonta et al. [95] in later experiments. As mentioned earlier, the long measurement times of LA-ICP-MS imaging experiments may cause significant changes in the instrument sensitivity. This was illustrated in an imaging experiment of a printed pattern with blue ink [95]. Blue ink contains copper, which was investigated as the analyte of interest. The pattern has been coated with a thin gold layer for use as a pseudo-internal standard. Features with equal amounts of ink deposition (i.e., copper concentration) measured at different time points were compared regarding the signal intensity. Figure 2 shows the signal of ^65^Cu with and without correction to the gold signal. During the 4.5-h measurement time, the absolute signal intensity for ^65^Cu decreased by 25 %, indicating a strong sensitivity drift. Normalization to gold as pseudo-internal standard corrects for this drift and keeps the sensitivity constant throughout the measurement time. Thus, the necessity and feasibility of signal normalization is underlined as the results show that an internal standard is necessary for reliable LA-ICP-MS imaging experiments.

In summary, quantitative determination of trace elements in biological tissues using LA-ICP-MS is still a challenging task, requiring extensive knowledge of sample composition and properties. Until now, no universal method has been established. Even though the preparation of matrix-matched standards seems to be the most straightforward method, some problems still remain. None of the other alternative methods are recognized as a reliable alternative to matrix-matched tissue standards because, as with every method, some limitations have to be considered.

### Electrophoretically separated metalloproteins

Besides the analysis of native soft tissues, also other soft sample materials can be analyzed using LA-ICP-MS. A major group of samples are electrophoretic gels. Separation of proteins or peptides in porous gel matrices is a commonly used bioanalytical method [101, 102]. The biomolecules can be separated on the basis of their mobility in a gel matrix, and visualized using staining methods. Subsequent identification (e.g., using mass spectrometry) can be performed. With the increasing knowledge on metalloproteins, metal detection within the separated proteins has also gained importance. Sensitive elemental analytical techniques that allow for direct analysis of the metals from the gels, like LA-ICP-MS, are a powerful tool to obtain quantitative information on the metal content after electrophoretic separation of the proteins. Quantification of metal contents in electrophoretically separated gels is significantly different from trace element quantification in biological tissues, as a result of the homogeneous composition of the separation gel. However, some crucial aspects also have to be considered to ensure reliable results. Micronebulization of an aqueous standard at the ablation site using either standards with natural isotopic composition [36], or isotopically enriched solutions [37], has been proposed for quantification. In those approaches, changes in ablation and/or ionization behavior of the analytes are not taken into account and therefore an approach with species-specific isotope dilution was proposed by Konz et al. [103]. While in all other methods, the standard is added after material ablation, this method relies on direct addition of the standard to the sample and provides the possibility to compensate for all changes that affect the protein of interest. However, the disadvantage of the method is the fact that the protein of interest has to be available in pure form with an isotopically enriched metal cofactor.

Because the thickness of the gels is typically in the order of 1 mm, the entire thickness is not ablated during a few laser shots. Therefore, the use of carbon as internal standard has been proposed [104]. Still, transferring the proteins to a membrane after separation (blotting) is used far more often, as this allows one to avoid direct analysis of the gel. The investigation of so-called Western blot membranes using LA-ICP-MS has been described in detail elsewhere [105, 106]. In contrast to tissue analysis, the variety of proposed methods in the field of protein analysis is smaller. As with tissue samples, thus far, no optimal quantification method has been found.

## Analysis of powdered samples

### Sample preparation

Most LA-ICP-MS applications in the fields of environmental research and life sciences focus either on obtaining bulk information (with high precision and accuracy) or on obtaining spatially resolved information, sometimes only semiquantitatively. In contrast to the compact samples discussed in the previous sections, powdered samples require some kind of pretreatment prior to LA-ICP-MS measurement. The approaches most frequently applied for the preparation of compact samples from powders include milling/grinding/sieving for sample homogenization, combined with pelletization [107–112], fusion to sample disks [29, 107, 113–115], or mounting/embedding [110, 116–119] of the sample in a polymeric resin. In some cases, alternative approaches such as ablating standard and sample in quick succession by placing them on a rotary platform or electroplating have been reported [28, 33, 120–125].

During pelletization, the sample powder is compacted using a hydraulic press. Usually, the sample is mixed with a binder to improve powder grain adhesion and to produce stable pellets. Also additives (in liquid or solid form) can be introduced for internal standardization, quantification (e.g., for standard addition or isotope dilution purposes), or to affect laser–sample interaction (i.e., increased energy absorption at the applied laser wavelength). To achieve a better homogeneity, the components are often milled or ground prior to mixing. The pressing step is usually optimized in terms of press power and pressing time. Depending on the ratio of binder to sample, target analytes may be diluted by up to a factor of 10. Thereby, matrix differences between samples will be reduced, but the detection power is decreased. Pelletization is a very easy approach without the need of high-tech equipment. The homogeneity achieved in pressed pellets is sufficient for most applications. However, in some cases the reproducibility achievable is constrained by the sample homogeneity.

For fusion, the sample powder is usually combined with lithium tetraborate or lithium metaborate or a mixture of both. At high temperatures (over 1000 °C), the sample is dissolved in the molten flux and, after cooling, very homogeneous fusion disks are obtained. Like with pelletization, additives can be introduced for internal standardization or quantification. Also by diluting with flux, the matrix similarity increases, while the sensitivity decreases. Fully automated fusion generators are available, reproducibly delivering homogenous samples and requiring only little analyst effort. Compared to pelletization, borate fusion offers a better homogeneity. However, fusion may be problematic for analytes with low boiling points (below 1000 °C), like As, Cd, or Zn. Analyte losses cannot be eliminated and therefore might give rise to systematic errors. Of course, with this type of sample preparation, information on Li and B as analyte elements is also lost.

The third approach that is frequently used is mounting/embedding. By mounting, the powdered samples are attached on an adhesive surface, like sticky tape or not completely dried epoxy resin. The surface can be coated before or after sample exposition to vary adhesive effects, or to introduce standards. Mounting is mostly used for qualitative and semiquantitative analysis. Adhesiveness is a limiting factor since the surface must be sticky enough to retain powder particles even after the nearby surface has been subjected to laser irradiation; total damage of the investigated particles or removal of particles next to the ablation site has to be prevented. The sample particulates can also be embedded completely in epoxy resin. After embedding, the resins usually need to be cut and polished prior to LA analysis. During preparation of the epoxy resins, standards and other supplements (e.g., surfactants for particle isolation) can also be added to improve the results. With both methods, information on individual particles can be obtained, which is not possible with pelletization or fusion. This aspect extends the possibilities of LA-ICP-MS (e.g., for 2-dimensional mapping), but the sample preparation is very tedious. However, as a result of the possibly widely different composition of the single particles, reliable quantification is practically impossible.

### Quantification strategies

#### Application of CRMs and in-house standards

Signal quantification using solid standards could be accomplished using either CRMs or in-house prepared standards. Usually, sample and standard need to be converted into a compact sample pellet or disk by one of the methods described in the previous section. Regardless of the sample preparation technique used, in all cases an element initially present in the sample and standard or added during sample preparation is used as an internal standard to correct for differences in ablation, transport, and ionization efficiencies. Concentrations of the internal standard in the sample and standard must be determined by complementary techniques (e.g., SEM-EDX, energy dispersive x-ray analysis) or must be sufficiently well known on the basis of stoichiometry.

Hondrogiannis et al. [108] used LA-ICP-TOF-MS to successfully classify 25 vanilla samples according to their origin. Three grams of vanilla powder was directly pressed into a sample pellet. External calibration was achieved versus NIST SRM 1549 (non-fat milk powder), NIST SRM 1575a (trace elements in pine needles), NIST SRM 1515 (apple leaves), NIST SRM 1547 (peach leaves), and NIST SRM 1570a (trace elements in spinach leaves). The method was validated using NIST SRM 1573a (tomato leaves). Eze et al. used LA-ICP-MS to investigate the composition of coal fly ash [33]. Fusion disks of each sample were prepared according to an automatic gas fusion procedure (Claisse M4 gas fusion instrument) with Claisse Flux as binder material. Quantification of 18 elements was achieved via external calibration versus NIST SRM 612 and using ^29^Si as an internal standard. USGS BCR-2 or BHVO 2G CRMs were used for method validation. Scarciglia et al. investigated soil and paleosoil samples [123]. Thin sections were prepared for LA-ICP-MS analysis and for calibration NIST SRM 612 was relied on because of the lack of soil CRMs. SiO_2_, quantified with SEM-EDX, was used as an internal standard. Relative standard deviations (RSDs) were less than 8 % for all elements and less than 5 % for most of them. Further applications include the analysis of Sahara dust samples [118], desert varnish [121], soil samples [116], biomass ashes [29], fly ash samples [113], ash related deposits [120], coral skeletons [125], and forensic investigations [110]. Detailed information can be found in Table 1.

**Table 1:** Pretreatment and quantification approaches for powdered samples

	Sample type	Sample preparation	Quantification	IS	Validation	Recovery	RSD	Ref.
Standard procedures	Vanilla samples	Pressed pellets	NIST SRM 1549, NIST SRM 1575a, NIST SRM 1515, NIST SRM 1547 and NIST SRM 1570a		NIST SRM 1573a	83–106 %	6.2–14.3 %	108
	Coal fly ash	Borate fusion	NIST SRM 612	Si	USGS BCR-2 or BHVO 2G			29
	Soil samples	Thin sections	NIST SRM 612	Si			<5–8 %	123
	Sahara dust samples	Adhesive tape	NIST SRMs 612 and GSD-1G	Si	USGS W-1 and BCR-2, MPI-DING T1-G and GSJ JG-1a	75–125 %	<15 %	118
	Desert varnish	Direct analysis	NIST 61X series		NIST SRM 612	80–120 %	<20 %	121
	Biomass ash	Borate fusion	NIST SRMs 610 and 612					33
	Fly ash samples	Borate fusion	NIST SRM 2691					113
	Ash related deposits	Embedded in epoxy resin	NIST SRM 2691					120
	Soil samples	Ashing, mounting in epoxy resin	NIST SRM 2691 and NIST SRM 1633b				15–40 %	116
	Coral skeletons	Glass-fused/cut into thin sections	NIST SRMs 610 and 612 and MPI-DING KL2-G	Ca	USGS MACS-1/NIST SRM 614	80–120 %	<4–15 %	125
	Forensic applications	Tape mounting and pelletization	USGS PACS-2, NIST SRM 2704, NIST SRM 2710 and NIST SRM 2710a	Sc, Lu			<15 %	110
	Furnace flue dust	Pressed pellets	Synthetic ZnO/Fe_2_O_3_ matrix	Rh	CRM 876-1, AG-6203, AG-6201, and AG-SX3705	85–115 %	<7 %	111
	Wood fibers	Pressed pellets	Pellets prepared with cellulose powder, softwood pulp					112
	Glass, silicate	Xerogel disks	NIST SRMs 610 and 612					32
	Aerosol samples	Direct analysis	Standard addition				<10–18 %	124
	Sunflower leaves	Direct analysis	Standard addition					73
	Compost samples	Pressed pellets	BCR-144R and CRM029-050 and standard addition				<10 %	109
Improved procedures	Various CRMs	Borate fusion	Isotope dilution		NIST SRM 1944, 2586, 2702, 2710a, 2711a, and 2780	95–120 %	<3 %	114
	Various CRMs	Pressed pellets, borate fusion	On-line isotope dilution		NIST SRM 610, 612, and 614, MESS-2 and PACS-2 and NIST SRM 2710a, 2711a, 1944, 2702, and 2780	85–110 %	3–21 %	107
	Oxide grains	Mounted on epoxy resin	On-line isotope dilution, ratio analysis		SRM U950a and U010 as well as natural uraninite grains		0.4–2.7 %	117
Specific approaches	Environmental samples	Borate fusion	Standard addition		NIST SRM 612	85–115 %	~10 %	115
	Carbonate	Adhesive tape	MPI-DING reference glasses		NIST SRM 610	90–110 %	1.6–24 %	119
	Soil & dust samples	Electroplating	Ratio analysis		NIST SRM 4353			122

If no suitable CRMs are available, or the range of analytes cannot be covered, the preparation of in-house standards is another possibility for quantification. Coedo et al. determined six elements in electric arc furnace flue dust [111]. Samples were pressed into pellets using paraffin and cellulose/*N*-butyl-methacrylate. Standards were prepared by spiking synthetic ZnO/Fe_2_O_3_ matrix (1:1) with multi-element solution standards and Rh as an internal standard. The approach was validated with four reference materials. Su et al. [112] determined the distribution of metals in single wood fibers. The fibers were fixed by pressing them onto pellets with graphite powder. For quantification, matrix-matched pellets were prepared with cellulose powder and softwood pulp, doped with multi-element standards. The difference in the amount of ablated material was compensated for by introducing a mass coefficient. Fitzpatrick et al. investigated sol-gel processes to establish in-house calibration standards [28]. They showed that S and Se can be added up to 3 % of the total xerogel concentration, while for transition metals the corresponding maximum level is 0.01 %. The xerogels thus obtained were used for calibration in the LA-ICP-MS analysis of NIST SRMs 610 and 612 (trace elements in glass), achieving satisfactory results with RSDs comparable to those achieved using glass CRMs. According to their work, xerogels seem to be a feasible alternative for glass CRMs. However, no accurate results could be produced for samples with high sulfide contents.

#### Improved standard approaches

Compared to the reported approaches using CRMs and in-house prepared matrix-matched standards, the quantification strategies can be even further improved by exploiting the concepts of single standard addition, multiple standard additions, or isotope dilution. Thus, remaining differences between sample and standard can be further compensated for, enhancing the quality of LA-ICP-MS analysis. Okuda et al. used LA-ICP-MS to analyze aerosol samples collected on cellulose nitrate filters [124]. The filters were directly ablated and 15 elements were investigated. Calibration was achieved by spiking the filters with standard solutions. Precisions better than 10 % RSD could be achieved, except for Al (11 %) and Cu (18 %).

da Silva and Arruda [73] prepared sample pellets for the measurement of Se and S in sunflower leaves, which were spiked with different amounts of the elements of interest. Different certified materials were used for validation of the method. Jiménez et al. [109] investigated compost samples for the presence of toxic metals. The samples were homogenized, ground, sieved several times, and pressed into pellets (200 mg). Quantification was achieved by external calibration versus matrix-matched standards (BCR-144R and CRM029-050) and standard addition with aqueous standards. The RSDs for quantification were better than 10 % for most elements. Particle size and therefore milling time were identified as factors having a high impact on the method precision.

If applicable for the target analyte(s), the addition of isotope-enriched spikes is also feasible, enabling analyte quantification using isotope dilution. This approach offers best results in terms of precision and accuracy, but is also expensive and limited to elements for which at least two isotopes can be measured interference-free. Malherbe et al. investigated the potential of this approach for the analysis of different CRMs. Prior to LA-ICP-MS measurement, sample disks were prepared by borate fusion [114]. NIST SRM 1944 (New York/New Jersey waterway sediment), NIST SRM 2586 (trace elements in soil containing lead from paint), NIST SRM 2702 (inorganics in marine sediment), NIST SRM 2710a (Montana I soil), NIST SRM 2711a (Montana II soil), and NIST SRM 2780 (hard rock minewaste) and a meteorite sample were analysed. The results obtained were in good agreement with the corresponding certified values and the precision was better than 3 % RSD for all elements investigated. For the analysis of powdered samples, the previously described concept of liquid standard nebulization is also used for quantification of LA-generated aerosols. Fernández et al. [107] proposed a quantification method with on-line double isotope dilution for a wide range of matrices. Samples were either analyzed directly (NIST SRMs 610, 612, and 614), pressed into pellets (USGS MESS-2 and PACS-2), or fused with lithium borate (NIST SRMs 2710a, 2711a, 1944, 2702, and 2780). The ablated aerosol of either standard or sample is mixed with the nebulized isotope-enriched spike solution or blank solution. The RSDs ranged from 6 to 21 % for pressed pellets and from 3 to 21 % for borate fusion. Lloyd et al. analyzed uranium oxide grains, retrieved from soil and dust samples [117]. The grains were mounted on epoxy resin; the latter was then ground and polished to access the interior of the grains. As a result of the lack of a suitable standard reference material containing ^236^U, quantification was achieved by introducing liquid reference materials NBL U950a (uranium particles) and NBL U010 (uranium isotopic standard) via a desolvating nebulizer. Natural uraninite grains were used as tertiary reference material to correct for mass bias. U isotope ratio analysis was implemented successfully with precisions of 0.4 % and 2.7 % RSD for ^235^U/^238^U and ^236^U/^238^U, respectively.

#### Specific approaches

Claverie et al. [115] proposed a new approach for quantification of six elements of environmental concern, e.g., in soil or sediment samples, using pellets fixed on a spinning platform. Samples as well as standards were prepared by lithium borate fusion. By placing standard and sample next to each other on a platform, which is spinning at a high speed during laser ablation, quasi-simultaneous ablation of sample and standard is achieved. The mixed ablation aerosols are analyzed and quantification is based on standard addition or isotope dilution. For five standard reference materials and meteorite rock, an average precision of 10 % RSD could be achieved. The experimental results obtained compared well with the corresponding certified values with maximum deviations of 15 %. Lu et al. [119] tried to overcome the need for an internal standard for LA-ICP-MS analysis of carbonate materials. Using an equation-based approach, the so-called MRM-NoIS calibration strategy, a successful quantification of different carbonate minerals was achieved by using four reference materials (NIST SRM 610, USGS MACS-3, USGS GP-4, MPI-DING). For trace elements RSDs of less than 10 % and for rare earth elements (REEs) and major compounds RSDs of less than 5 % were observed. Cizdziel et al. investigated plutonium in US soil and dust samples [122]. Pu was spiked with a tracer, leached from the sample, and extracted from the leachate by anion exchange chromatography. Afterwards, the recovered analyte was electroplated on a stainless steel planchette disk for LA-ICP-MS analysis. LA-ICP-MS results were compared with liquid ICP-MS results, which were validated using NIST SRM 4350b (river sediment, radioactivity), NIST SRM 4353 (rocky flats soil number 2), and IAEA 385 (radionuclides in Irish Sea sediment). The authors reported a successful fingerprinting of Pu in soil and dust samples with LA-ICP-MS.

### Figures of merit

Since detection limits and data for the level of reproducibility achieved have not been published in any of the reviewed contributions, this section aims at providing a comparison between the methods, accounting for all special applications. Besides the approach used for preparation of compact samples, the quantification strategy also has to be taken into consideration. Furthermore, sample homogeneity is another limiting factor (especially for pressed pellets). Also, the concentration ranges of the target analytes as well as the instrumentation applied influence the quality of the results obtained.

In general, detection limits (LOD) were found to vary between several micrograms per kilogram and some milligrams per kilogram, depending on the instrumentation used and the analyte of interest. With pelletization, LODs around 0.035 mg kg^−1^ were obtained for different elements in vanilla samples [108]; for compost samples [109], values ranging from 0.01 to 0.8 mg kg^−1^ were reported. For fusion, the LODs varied from 0.02 to 4 mg kg^−1^ [114] with SF-ICP-MS and Q-ICP-MS. For tape mounting analysis and subsequent LA-ICP-MS analysis using SF-ICP-MS, detection limits from 0.3 μg kg^−1^ to 10 mg kg^−1^ were observed [118]. LODs varying from 0.001 to 0.5 mg kg^−1^ were reported for direct analysis of the sample without pretreatment [121].

In contrast to sensitivity, the reproducibility of a measurement is less dependent on the MS instrumentation used. Overall, reported RSDs were in the order of less than 3 to 50 %. The pelletization approach resulted in measurement reproducibilities varying between 6 and 21 % for the elements Pb, Rb, and Sr [107], and 6 to 14 % for 11 elements in vanilla [108] and less than 15 % for 12 elements in soil [110]. Fusion approaches showed comparably lower RSDs, a result which could be attributed to the improved sample homogeneity obtained with this approach. Published results vary between less than 3 % [114], 3 and 21 % for Pb, Rb, and Sr [107], and 10 % for six elements in environmental matrices [115], depending on target element and calibration strategy. For applications using the mounting/embedding approach for sample preparation, RSDs ranged from 15 to 50 % for the halogens Cl, Br, and I in ashed soil samples [116], and 15 to 25 % for 60 elements in dust samples using fs-LA-ICP-MS [118]. Poorer RSDs often result from the low analyte signals observed when analyzing single particles, which give rise to very small amounts of ablated material only.

## Dried droplet analysis of liquid samples

As demonstrated in the previous sections, LA-ICP-MS is a versatile tool for solid sampling, suited for both bulk analysis and for mapping analyte distributions, as well as for combinations thereof. Consequently, the vast majority of samples being analyzed by LA-ICP-MS today are solids, especially since LA-ICP-MS circumvents the sometimes cumbersome digestion procedures otherwise required. For liquid samples and sample solutions, sample introduction in ICP-MS analysis is traditionally accomplished using a nebulizer. Pneumatic nebulizers are available in numerous modifications to suit practically any kind of liquid matrix [126]. However, heavily matrix-loaded liquid samples, such as urine or blood, present a challenge even for the most matrix-tolerant nebulizers. Such demanding matrices require at least dilution or partial digestion, which results in an increased workload. If high sample throughput is required, alternative sample introduction methods are therefore necessary.

As an alternative to pneumatic nebulization, laser ablation of dried liquids offers the aforementioned matrix-tolerance and sample throughput. In this section, the concept and some practical aspects of dried droplet laser ablation will be discussed. The performance of the method, as well as instrumental limitations will be highlighted, and a comprehensive overview of the related literature, including application examples, will be given.

### The concept of dried droplet laser ablation

The basic concept of dried droplet laser ablation consists of depositing a well-defined volume of liquid sample on a solid support, evaporating the solvent, and examining the remaining dried residue by means of LA-ICP-MS. To unmistakably state that only the dried residue of a liquid sample is being analyzed, the term “dried droplet laser ablation” will be used throughout. To the best of our knowledge, Yang et al. introduced this method to ICP-MS in 2005 [127]. The present review will focus exclusively on the ablation of dried liquid samples, although it has been demonstrated that direct liquid ablation is also possible [128, 129].

As simple as the concept of dried droplet laser ablation may seem, its implementation can hold some pitfalls. One risk is to compromise the natural homogeneity inherent to the liquid sample. Method development in dried droplet laser ablation should therefore aim at preserving the sample’s original elemental composition throughout the analytical process, or at providing a sound strategy to compensate for any inhomogeneities introduced artificially during sample preparation. There are some methods reported in the literature that appear to be similar to dried droplet laser ablation in the sense that some part of a liquid sample is dried and analyzed by laser ablation. However, with those methods, the homogeneity of the liquid sample is abandoned by design. Hence, it is difficult or impossible to obtain quantitative information. Such methods are, for example, the combination of thin-layer chromatography with laser ablation [130–134] or the analysis of substrates which are immersed in a sample, removed from the liquid, and subsequently dried [135, 136].

The motivation for using dried droplet laser ablation instead of more established sample introduction techniques is in all cases reported to be (a combination of) the following four features: coverage of (sub-)microliter sample volumes, while offering (sub-)microgram per liter detection limits in case of ICP-MS detection, removal of solvent to allow coupling ICP-MS as an element-specific detector to chromatographic systems and to obtain less polyatomic interferences arising from the solvent, simplification of sample logistics, as well as direct sampling of challenging liquid matrices. Although some of these features could also be achieved with alternative solid sampling methods, such as solid sampling graphite furnace AAS or electrothermal vaporization (ETV) ICP-MS, dried droplet laser ablation offers two significant advantages over graphite furnace techniques. First, it is a genuine multi-element technique as opposed to conventional AAS, or compared to high-resolution continuum source AAS with limited multi-element capabilities. Secondly, LA allows complete desorption of the sample, whereas in ETV carbide formation may hamper correct quantification (e.g., [137, 138]). Such problems are not observed with LA [139]. In some cases, sample throughput was found to be higher with dried droplet laser ablation than with ETV-ICP-MS [139] but this certainly depends on the measurement protocols deployed and cannot be generalized.

### Preparation of dried droplets

As stated above, the key aspect of dried droplet laser ablation is to preserve the inherent homogeneity of the liquid sample throughout sample preparation and measurement. If an artificial inhomogeneity is newly introduced, this should be done in a reproducible way, in order to be able to fully compensate for it. From everyday experience, it is well known that dried residues, e.g., coffee stains in the kitchen, are usually far from being homogeneously shaped. It is the scope of this section to provide some very basic physical insights into the processes involved in droplet-drying, although the literature on this topic is vast and the interested reader is referred to the numerous specialized reviews. Three parameters play a major role in terms of dried droplet shape: (a) choice of the surface used for droplet deposition, (b) drying conditions, and (c) the matrix of the liquid sample:

- When drying droplets on hydrophilic surfaces, ring-shaped residues are frequently obtained. This “coffee stain effect” was described by Deegan et al. [140, 141] to be caused by a radial flow, which transports liquid from the core of the droplet to its perimeter, where the solvent evaporates more easily. This radial flow is the consequence of one precondition inherent to this physical model: the contact line (the perimeter of the droplet) does not shrink during the drying process. Several authors provided experimental data to support this model (e.g., [142–144]). Contrarily, in the case of a hydrophobic surface, deposition of concentric rings or small spots in the center of the droplet can be observed [145–147], as the contact line continuously or periodically shrinks while drying.
- In addition to the flow patterns that lead to ring deposition, the interaction of dissolved or colloidal matter with the contact area, as well as convective currents can contribute to the dried pattern [144, 148]. Such convective currents are related to the drying rate, which in turn depends on temperature, relative humidity, and heat conductivity of the substrate [144, 149, 150]. Hence, the experimental setup might also influence the shape of the dried residue.
- Finally, the matrix of the sample (e.g., salt or protein concentration) also influences the shape of the dried residue [151, 152]. In the first publications that describe dried droplet laser ablation, small sample aliquots of 20 μL were pipetted onto hydrophobic polystyrene plates and dried under ambient conditions [127, 139, 153]. Owing to differences in the matrix (purely inorganic salts in the case of standards, with organic constituents in case of samples), the shape and size of the dried residue depended strongly on the sample matrix [127].

**Fig. 3:**
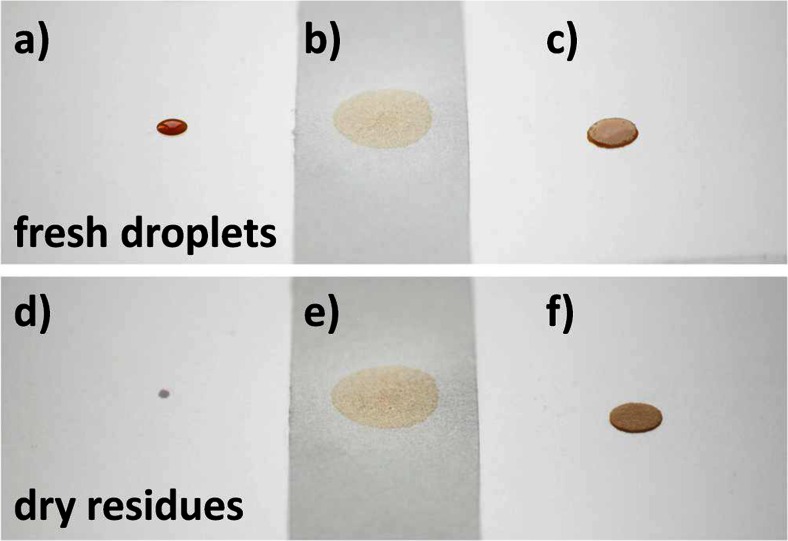
Strategies for sample application in dried droplet LA analysis. Deposition of a defined sample volume on hydrophobic surfaces (**a**), filter paper with dimensions much greater than droplet diameter (**b**), and confined, circular, hydrophilic areas with diameter of circular area no greater than droplet diameter (**c**). Dried residues after evaporation of the solvent on a hydrophobic surface (**d**), filter paper (**e**), and precut filter disks of filter paper (**f**)

In view of the aforementioned three parameters, a drying droplet is a complex system and it is easy to understand that the morphology of the final dried residue is difficult to predict. Although the references provided show that it is indeed possible to control the morphology of the dried residue, such experiments are most likely beyond the scope of analytical laboratories. However, it is possible to minimize the influence of those factors, which have the most pronounced effect. As the choice of the solid substrate plays a major role in terms of droplet morphology, this factor was considered and optimized in most reported cases of dried droplet laser ablation. The following three types of solid substrates were applied: 1. hydrophobic surfaces, 2. filter paper (paper diameter much greater than droplet diameter), and 3. confined, circular, and hydrophilic areas (diameter of circular area no greater than droplet diameter). For visualization, examples of these approaches are provided in Fig. 3.

#### Method I

Using a hydrophobic surface for droplet deposition results in a small dried residue, as described above. Typical diameters of dried residues are around 100–1600 μm, depending on droplet volume and sample matrix (Table 2). As the droplet shrinks continuously, the coffee stain effect will be observed only at a very late stage of the drying process, or not at all. Hence, it is straightforward to ablate the entire dried residue with only a few laser pulses. Hsieh et al. [154, 155] demonstrated that this approach allows for external calibration when quantifying metals in whole blood. Yet, as other authors have found, the extreme preconcentration on a small spot has the disadvantage of pronounced matrix effects by co-existing sample constituents. For example, Yang et al. [127] showed significant signal suppression by sodium present in the sample. On the other hand, deposition of droplets on a substrate that enhances sample ablation as a result of strong interaction with the laser light has been demonstrated [156, 157], in combination with automated deposition of liquids. Table 2 summarizes the literature that applied the approach of hydrophobic surfaces.

**Table 2:** Method I: droplet deposition on hydrophobic surface

Sample matrix	Droplet volume	Size of dried residue	Sample loading^a^	Surface material	LOD	Reproducibility	Additional information	Ref.
Drinking water, yeast extract	20 μL	150–500 μm	10^−4^–10^−3^ μL μm^−2^	PS	0.08–0.12 ng mL^−1^ (aqueous), 0.06–0.09 ng mL^−1^ (standard addition), 0.05–0.08 ng mL^−1^ (isotope dilution)	4.6–23 % RSD (aqueous with I.S., *n* = 4), 4.7–8.2 % RSD (standard addition with I.S., *n* = 4), 3.5–3.9 % RSD (isotope dilution, *n* = 6); 0.5 % RSD (Se in yeast, isotope dilution, *n* = 5)	NaAc matrix was added to water samples	[127]
HPLC fractions of yeast extract	20 μL	600 μm	7 × 10^−5^ μL μm^−2^	PS	36–110 μg g^−1^ (Se)	0.55–0.77 % RSD (species-specific isotope dilution)	No matrix required due to high salt load of samples	[153]
Digested biological tissue, nearshore seawater, and river water	20 μL	100 μm 1.6 mm	10^−5–^3 × 10^−3^ μL μm^−2^	PS	0.033 pg mL^−1^ (Pu), 0.051 pg mL^−1^ (Th), 0.072 pg mL^−1^ (U).	8 % RSD (with I.S., *n* = 10)	Chromogenic matrix investigated	[137]
Cr species via capillary electrophoresis	100 nL	100–500 μm	5 × 10^−7^–10^−5^ μL μm^−2^	PETG	0.2–6.5 μg L^−1^	Below 3 % RSD	α-Cyano-4-hydroxycinnamic acid	[156]
Blood reference materials	0.5 μL	700–900 μm	8 × 10^−7^–10^−6^ μL μm^−2^	Other	0.1 ng mL^−1^	Below 10 % RSD for all samples	Methylene blue as indicator and to improve ablation yield	[154]
Cell cultivation medium and cell lysate	<20 nL	<300 μm	3 × 10^−7^ μL μm^−2^	PETG	26 fg Cu (100 nL droplet, therefore 26 ng L^−1^)	5 % RSD under optimized conditions for samples	Rhodamine B added for visibility	[157]
Cr species via liquid-liquid micro extraction, synthetic seawater	7 μL	5 mm	4 × 10^−7^ μL μm^−2^	PS	0.11 μg L^−1^	4–8 % RSD	Organic matrix, internal standard	[169]
Various “meat” reference materials (oyster tissue, etc.)	50–100 μL	1 cm	6 × 10^−7^–10^−6^ μL μm^−2^	PTFE, PS	0.05–6 μg kg^−1^ dry mass (corresponds to 1.25–240 ng L^−1^)	5–10 % RSD	No additive, organic digest	[170]
Mineral water, tap water, swimming pool water, and water from two artificial lakes	1 μL	600 μm	4 × 10^−6^ μL μm^−2^	PTFE	0.05–0.81 ng mL^−1^	~5 % RSD (for *n* = 3, recovery experiment)	Methylene blue added for visibility	[171]
Seronorm blood reference material	0.5 μL	–	–	PTFE	0.14–29 ng mL^−1^ (Be–Mg)	6 % RSD within-run precision, 4–8 % RSD between-run precision	Methylene blue added for visibility	[155]
SLRS-4 river water reference material, lake water, and synthetic seawater	1 μL	480–850 μm	2 × 10^−6^–6 × 10^−6^ μL μm^−2^	PTFE	0.03–0.2 pg mL^−1^ (enrichment factor 32)	2–5 % RSD	Methylene blue added for visibility	[172]
Human urine from Fabry disease patient and control	1 μL	–	–	PTFE^)^	0.003–0.58 μg g^−1^	<20 % RSD	Spiked samples for calibration	[173]

*PS* polystyrene, *PETG* poly(ethyleneterephthalate)glycol, *PTFE* polytetrafluoroethylene, *Other* “hydrophobic filter membrane”

^a^Sample volume/area of dried residue (assuming a circular spot)

#### Method II

Applying a droplet on a large piece of filter paper makes sample preparation very easy, as the liquid is immediately absorbed by capillary action [158, 159]. Once dried, the residues can be easily transported and stored as the analyte is incorporated in the paper fibers [160]. Consequently, blotting cards are widely used in clinical settings [161], e.g., in collection of blood samples. Yet, even if the dispensed volume of sample is well defined, differences in viscosity can lead to a different migration behavior on the paper, combined with chromatographic effects [162, 163]. Careful design of laser ablation patterns is therefore required. Table 3 gives an overview of papers that report on analysis of “freely migrated” droplets on filter paper, via LA ICP-MS. Also, typical diameters of dried residues and applied sample volumes are given.

**Table 3:** Method II: droplet deposition on large filter paper sheets

Sample matrix	Droplet volume	Size of dried residue	Sample loading^a^	Surface material	LOD	Reproducibility	Additional information	Ref.
Blood spotted on paper, from a lab proficiency test	–	–	–	Whatmann filter paper	0.9 μg dL^−1^ (Pb)	7 % RSD (in-between droplets and also within droplet)	Sample directly spotted without any other treatment	[158]
Co in a drug preparation, Pb in whole blood, and Sn in food samples	500 nL	–	–	Filter paper with additive	1–60 ng L^−1^	10 % RSD (spot-to-spot)	Different additives to improve laser yield	[168]
Pb and Cd in BCR-634 whole blood reference material	200 pL	–	–	Filter paper with additive	0.5 pg Pb 0.02 pg Cd (equal to 2.5 and 0.1 ng L^−1^ with 200 nL of sample)	25 % for Pb and 8 % for Cd with standard solutions using ^13^C as internal standard, for samples: 5 % for Pb and 35 % for Cd	Repeated deposition of 65 pL droplets, ablation of several droplets at the same time	[167]
Blood (reference materials and real samples)	5 μL	5–6 mm	3 × 10^−7^ μL m^−2^	Filter paper	0.040–0.054 μg L^−1^	3–9 % RSD (quantitative) 1500 ppm (isotope ratios)	Analysis vial split aerosol-flow (single-collector/multi-collector ICP-MS)	[159]

^a^Sample volume/area of dried residue (assuming a circular spot)

#### Method III

By providing a hydrophilic area with a clearly defined border, samples can only migrate within this area. Therefore, samples with low viscosity or challenging matrix are confined on the same area as purely aqueous samples. This approach was used by Choi et al. [164] to analyze photo-resist, deposited on micro-machined polymer pillars. A somewhat different approach was presented by Aramendía et al. [160] who applied precut disks of filter paper on a hydrophobic surface. By doing so, large volumes of sample could be applied on a small area, thus enhancing sensitivity 10-fold [160]. However, the coffee stain effect is very pronounced with this type of sample preparation, since the precut filter disk is the ideal model of a fixed contact line (as described above, a fixed contact line results in a constant droplet area during the drying process. As the evaporation on the rim of the droplet is faster than in the center of the droplet, a liquid flow is created which transports material to the rim of the droplet which is the origin of the coffee stain effect). Crater-shaped analyte distributions were therefore obtained [18, 160, 165]), as also observed in MALDI-MS [166]. Table 4 gives an overview of the related literature and typical sample loadings (microliters per millimeter squared).

**Table 4:** Method III: droplet deposition on confined, circular, and hydrophilic areas

Sample Matrix	Droplet volume	Size of dried residue	Sample loading	Surface material	LOD	Reproducibility	Additional information	Ref.
Photo-resistant used in photolithography	64.7 pL	150 μm	4 × 10^−6^ μL μm^−2^	PDMS-columns (micro-machined)	2.33, 15.4, 5.72 ng mL^−1^ (Al, Cu, Pb)	17.1–46.9 % RSD (due to extremely low sample volume)	No matrix added, photo resist	[164]
Human urine from supposedly healthy patients	300 μL	16 mm	10^−6^ μL μm^−2^	Filter paper (precut saturated filter disks)	0.1–13 μg L^−1^	2–5 % RSD	No additive	[160]
Cu isotopes in urine of Wilson’s disease patients, treated patients and one control patient	300 μL	16 mm	10^−6^ μL μm^−2^	Filter paper (precut saturated filter disks)	–	200–500 ppm RSD intra-spot, and 540 ppm RSD inter-spot	No additive, corona ablation with 10 kHz	[18]
Phosphorus in fermentation media	10 μL	5 mm	5 × 10^−7^ μL μm^−2^	Filter paper (precut saturated filter disks)	10 μg mL^−1^ (ICP-OES detection)	10 % RSD	Analysis via laser ablation ICP-OES	[165]

^a^Sample volume / area of dried residue (assuming a circular spot)

### Quantification approaches, sensitivity, and reproducibility

The quantification process also needs to be adapted to the approach used for sample preparation. The sensitivity is influenced to a large extent by the analyte loading, i.e., the amount of sample per unit area (e.g., microliters per millimeter squared). In Tables 2–4, this value can be compared for the three application modes (methods I–III), and in general, highest analyte loading is observed with method I. However, as discussed above, matrix effects are most pronounced with this approach; therefore, lower analyte loadings (method II or method III) can be beneficial, depending on the individual analytical setup and sample type.

In the case of sample preparation on a hydrophobic surface (method I), the small residue can be ablated completely using spot, grid, or line patterns. Thus, the entire signal is collected in a short time, giving rise to a sensitivity which is comparable to that achievable with conventional nebulizer systems. Yang et al. [127] even demonstrated a 2–7-fold improved absolute sensitivity (counts per nanogram) compared to pneumatic nebulization when analyzing aqueous standard solutions. This finding is also due to the fact that the transport efficiency of laser ablation systems is superior to that of pneumatic nebulizer systems [127]. The general sensitivity of dried droplet laser ablation in combination with hydrophobic surfaces obtained in practice can therefore be expected to be similar to that with pneumatic nebulization (see Table 2).

Partial ablation of freely deposited droplets (method II) might result in erroneous results, as demonstrated in [160], unless closely matrix-matched standards are used [158]. The reason for this is that sample-to-sample variations in terms of viscosity result in different sample spread across the filter. The complete consumption of the dried residue [159] is a feasible way to avoid this problem, but requires specialized laser equipment. If the deposited volumes are very small [167, 168], quantitative ablation from filter paper becomes easy, especially in the presence of substances that improve the laser ablation yield, such as black ink deposited prior to the droplet [167, 168].

In the case of uniform sample geometry due to circular hydrophobic areas (method III), the coffee stain effect is very pronounced, as discussed above. Although a quite homogeneous analyte distribution is obtained in the center of the droplet, the extent of ring formation will depend on the sample matrix. Therefore, standard addition or isotope dilution is required if the laser is focused onto the center of the filter disk. In a recent publication, Nischkauer et al. [165] showed that the bias resulting from this centrosymmetric distribution of analytes can be easily compensated for. Instead of performing laser ablation only in the center [160] or only in the rim [18] of the precut filter disk, it was proposed to perform radial line scans that pass across the entire sample, including the center. The resulting U-shaped signal was then integrated and was found to be proportional to the concentration in the initially liquid sample, without the need to ablate the entire filter disk, and without the need for matrix-matching or the use of an internal standard [165].

The reproducibility of dried droplet laser ablation is intrinsically compromised, compared to pneumatic nebulization, as a result of the additional error introduced by repeated droplet deposition and by the transient signal mode. Interdroplet reproducibilities range between 3 % and 23 % RSD for samples measured directly, with typical values ranging between 3 % and 10 % RSD [127, 137, 154, 155, 157, 158, 160, 165, 167–173]. When automated, droplet deposition can be achieved with greater precision [174, 175], but the higher uncertainty inherent to solid sampling techniques will most likely persist. In case of isotope dilution and isotope ratio measurements, better reproducibility was reported (540 ppm RSD interspot [18], 0.55–0.77 % RSD for species-specific isotope dilution [153]) than in the case of pure quantitative measurements.

## Conclusions

Although geoscience is still the main field of application of LA-ICP-MS, its use in the fields of environmental research and life sciences increased continuously during the last few years. High sensitivity combined with excellent spatial resolution is the main reason for using LA-ICP-MS in the analysis of hard and soft tissues, as well as of powdered environmental samples. The capabilities for performing imaging studies or isotope ratio measurements are additional advantages of LA-ICP-MS. The applications published so far cover a wide range of sample matrices, target analytes, and concentration ranges. Nevertheless, they have one common problem—reliable quantification. The strategies used to circumvent the influence of elemental fractionation and matrix effects in LA-ICP-MS analysis, which are considered as the main problems hampering reliable quantification, are rather similar, although the resulting interferences differ between individual applications. Table 5 presents a compilation of the most frequently applied approaches for quantification, indicating that the use of in-house prepared standards in combination with an internal standard is the dominating strategy. Improved concepts for sample preparation as well as for application of matrix-matched standards will further enhance the potential of LA-ICP-MS for the analysis of environmental, biological, and biomedical samples. The choice of an appropriate internal standard is still of major concern in many applications, especially when no sample-inherent element is available. Thus, further methodological developments are required, e.g., in the case of tissue analysis the application of polymeric layers or thin metal coatings has been shown to be promising. Special attention should be paid to the application of fs-laser systems, which offer distinct improvements in terms of matrix effects and elemental fractionation. Another prerequisite for the acceptance of LA-ICP-MS as an alternative to traditional procedures for the quantitative determination of trace elements is the availability of appropriate reference materials. Especially for life science applications, the development and production of a larger range of CRMs is highly desirable. In contrast, for environmental samples (soil, fly ash, dust, etc.) a wide variety of CRMs is available. However, as those materials have been designed for liquid analysis after mineralization, none of them is applicable for direct LA-ICP-MS analysis because of their inhomogeneity on the microscale. Particularly considering the comparability of measurement results, the availability of at least a couple of compact standard materials with sufficient homogeneity should be aspired to.

**Table 5:** Selection of frequently applied procedures for signal quantification in LA-ICP-MS analysis

Quantification approach	Biogenic carbonates	Hard tissues	Soft tissue	Powdered samples	Liquid samples
CRM/SRM	27, 38–48, 50–54	58–62		108, 118, 121, 123	154
In-house prepared standards					
Non matrix-matched standards					
Use of well-characterized materials	43	64–68			158
Thin films on sample or substrate			94		
Gelatin, agarose gel, sol-gel standards			91, 92, 93	32	
Printed pattern			95, 96		167, 168
Dried droplets (aqueous standards)					127, 138, 154, 157
Matrix-matched standards					
Preparation of pellets	69			71, 109, 111, 112	
Fusion to disks	31, 32			29, 33, 113–115	
Embedding into polymer resin			97	116, 120	
Homogenized tissues			90		
Matrix-adjusted dried droplets					155, 164, 169–173
Specific approaches		63, 66, 67			
Nebulized liquid standards					
Calibration/standard addition	64, 65		88, 89	117	
IDMS			89, 103	107	
Internal standard correction					
Sample-inherent element	27, 28, 38, 39, 43, 46	58–67	75, 104	29, 118, 123, 125	
Homogeneously spiked to the sample			90, 97	110, 114	127, 139, 160, 169
Applied as thin layer on/below sample			79, 94, 95, 100		160
On-line addition of dried aerosol			88, 89		
